# The Role of Mechanical Force and ROS in Integrin-Dependent Signals

**DOI:** 10.1371/journal.pone.0064897

**Published:** 2013-05-30

**Authors:** Kathrin S. Zeller, Anjum Riaz, Hamid Sarve, Jia Li, Anders Tengholm, Staffan Johansson

**Affiliations:** 1 Department of Medical Biochemistry and Microbiology, Uppsala University, Uppsala, Sweden; 2 Centre for Image Analysis, Swedish University of Agricultural Sciences, Uppsala, Sweden; 3 Department of Medical Cell Biology, Uppsala University, Uppsala, Sweden; University of Birmingham, United Kingdom

## Abstract

Cells are exposed to several types of integrin stimuli, which generate responses generally referred to as “integrin signals”, but the specific responses to different integrin stimuli are poorly defined. In this study, signals induced by integrin ligation during cell attachment, mechanical force from intracellular contraction, or cell stretching by external force were compared. The elevated phosphorylation levels of several proteins during the early phase of cell attachment and spreading of fibroblast cell lines were not affected by inhibition of ROCK and myosin II activity, i.e. the reactions occurred independently of intracellular contractile force acting on the adhesion sites. The contraction-independent phosphorylation sites included ERK1/2 T202/Y204, AKT S473, p130CAS Y410, and cofilin S3. In contrast to cell attachment, cyclic stretching of the adherent cells induced a robust phosphorylation only of ERK1/2 and the phosphorylation levels of the other investigated proteins were not or only moderately affected by stretching. No major differences between signaling via α5β1 or αvβ3 integrins were detected. The importance of mitochondrial ROS for the integrin-induced signaling pathways was investigated using rotenone, a specific inhibitor of complex I in the respiratory chain. While rotenone only moderately reduced ATP levels and hardly affected the signals induced by cyclic cell stretching, it abolished the activation of AKT and reduced the actin polymerization rate in response to attachment in both cell lines. In contrast, scavenging of extracellular ROS with catalase or the vitamin C analog Asc-2P did not significantly influence the attachment-derived signaling, but caused a selective and pronounced enhancement of ERK1/2 phosphorylation in response to stretching. In conclusion, the results showed that “integrin signals” are composed of separate sets of reactions triggered by different types of integrin stimulation. Mitochondrial ROS and extracellular ROS had specific and distinct effects on the integrin signals induced by cell attachment and mechanical stretching.

## Introduction

Integrins are transmembrane cell-matrix receptors able to act as signaling mechano-sensors by linking extracellular ligands to actin filaments in adhesion sites. [1]–[3]. Several integrin adhesion site-associated proteins can change conformation upon mechanical stimulation and thereby expose cryptic binding or phosphorylation sites, three prototype examples being talin, p130CAS, and fibronectin (FN) [4]–[6]. Moreover, mechanical force has been reported to regulate the structure and function of integrins [7]. However, integrins generate signals also upon mere clustering [8], and ligand binding induces structural changes in integrins that may trigger additional signals [8], [9]. Although integrins have been extensively studied, it is still unclear what the specific signaling responses are during the different types of integrin stimulation, e.g. cell attachment, contractile forces generated within cells, or external forces acting on cells.

The signals generated during cell attachment have been shown to be strongly affected by integrin-triggered production of reactive oxygen species (ROS) from several sources. ROS derived from mitochondria [10], [11] as well as from NADPH oxidases (NOXes) and 5-lipoxygenase (5-LOX) [12], [13] were reported to have important roles in integrin-mediated attachment, spreading, and the associated changes in the cytoskeleton. Taddei et al. [11] proposed that mitochondrial ROS are involved in an early phase during cell attachment, whereas 5-LOX would mainly be responsible for the later phase of ROS generation.

ROS involvement has been implicated also in reactions induced during cell stretching by external force. Less is known about the involved receptors and the site of ROS production in response to this stimulus; NOXes [14], [15] as well as mitochondria [16] have been suggested as ROS sources, and cross-activation between different sources has also been reported [17]. Since ROS react promiscuously with numerous biomolecules, the specificity of their influence on cellular signaling events is believed to depend on the site of production [18] and on the modification of particularly redox-sensitive molecules. Examples of such targets range from receptors, phosphatases [19] and kinases [20] to actin [21], [22], actin-associated proteins [23]–[25] and transcription factors [20], [26]. ROS also regulates small GTPases in a complex fashion: depending on the context, ROS can both indirectly and directly activate or inhibit these proteins. For example, RHOA can be activated independently of GEFs by oxidation of critical cysteine residues [27]. Furthermore, RAC1 is required for the activation of several NOXes [28], which in turn are involved in the down-regulation of RHOA activity via LMW-PTP and p190RHO-GAP [29], [30].

In spite of an impressive amount of collected data on the roles of ROS during integrin signaling, many observations have to be judged with caution. The physiological relevance of results obtained by addition of unphysiological concentrations of reactive compounds such as hydrogen peroxide to mimic endogenous ROS production, or N-acetyl-cysteine to scavenge ROS can be questioned [20], [31], and there are serious concerns about the selectivity of the available oxidase inhibitors. Furthermore, several methods to measure ROS levels have been shown to actually generate ROS [31].

In the present study we aimed to clarify the specific signaling responses to different integrin stimuli by comparing the effects induced after a) ligand binding during initial cell attachment, with and without the inhibition of intracellular contraction and b) cyclic cell stretching. In addition, the effects of ROS in the response to these different integrin stimuli was investigated with emphasis on avoiding reportedly unspecific oxidase inhibitors and other reagents that are prone to artifacts [31].

## Materials and Methods

### Cells

The following cell lines were used: GD25 cells (β1-integrin knock-out mouse fibroblasts) and GD25β1 cells (GD25 cells re-expressing mouse β1 integrin) have been described [32]. BJ hTERT cells (human foreskin fibroblasts immortalized by forced expression of human telomerase reverse transcriptase) [33] were kindly provided by Paraskevi Heldin (The Ludwig Institute for Cancer Research, Uppsala). BJ hTERT cells were chosen for these studies because of their non-transformed phenotype. All cells were maintained in DMEM containing Gluta-Max™^−^I and pyruvate (Invitrogen, #31966047) supplemented with fetal bovine serum. The medium for the GD25β1 cells additionally contained 20 µg/ml puromycin.

### Antibodies and other reagents

The following antibodies were obtained from Cell Signaling Technology, USA: anti-AKT-pS473 (#9271), anti-p44/42 MAPK-pT202/pY204 (pERK1/2) (#9106), anti-p130CAS-pY410 (#4011), and anti-myosin light chain 2-pS19 (#3671). Anti-cofilin-pS3 was from Sigma-Aldrich (C8992), anti-MYPT1-pT853 (sc-17432), and HRP-conjugated anti-actin (sc-1616) were from Santa Cruz Biotechnologies. HRP-conjugated donkey anti-rabbit (NA9340) and HRP-conjugated sheep anti-mouse (NA9310) were from GE Healthcare, UK. The ECL kit (#RPM2106) was from GE Healthcare, Uppsala.

Rotenone was purchased from Dr. Ehrenstorfer GmbH (Germany, C16820000), sodium azide (#6688) from Merck, and 2-deoxy-D-glucose (D8375), superoxide dismutase (S5395), catalase (C1345, 2–5 kU/mg; calculated with 3 kU/mg) and the vitamin C derivative L-ascorbic acid 2-phosphate sesquimagnesium salt hydrate (Asc-2P) (A8960) were from Sigma. Since Sigma does not state how much water Asc-2P actually contains, but provides the information that it is usually 5 to 7 moles water per mole compound, the used Asc-2P concentration in this study should be within the range of 350 µM (7 moles water/mole Asc-2P) to 380 µM (5 moles water/mole Asc-2P). Y27632 was obtained from TOCRIS (#1254), Blebbistatin from Cayman Chemicals (#13013), and the cyclic RGD peptide (Arg-Gly-Asp-D-Phe-Lys) for inhibition of αvβ3 and αvβ5 integrins was from Peptide International (Kentucky, USA). Hydroethidine (HE, or dihydroethidium, DHE) was from Invitrogen (D11347), and xanthine (X0626) and xanthine oxidase (X4376) were from Sigma.

BSA fraction V was bought from Roche and Pluronic® F108NF Prill Poloxamer 338 (PL) from BASF. Invasin, FN and vitronectin (VN) were produced as described previously [34]–[36]. Elastosil M4601 (Wacker) used for casting the silicon chambers was obtained from Abic Kemi, Norrköping, Sweden. The silicon foil used as bottoms for the stretch chambers was from the Bisco Silicone Line (Rogers Cooperation, USA; HT-6240, 0.25 mm thickness) and obtained from Lebo Production AB, Stockholm, Sweden.

The colorimetric ATP assay kit (#K354-100) and the total antioxidant capacity kit (#K274-100) were from BioVision.

### Cell adhesion assay

Wells of a 96-well plate were coated with invasin, FN, VN or PL, and subsequently any remaining uncoated surface was blocked with PL [32]. GD25β1 or BJ hTERT cells were harvested by trypsin-EDTA, and the trypsin was inactivated by culture medium. The cells were then washed with serum-free DMEM and seeded in DMEM with or without the indicated amount of cyclic RGD peptide. After 60 minutes, non-attached cells were removed and the amount of attached cells was determined after staining with 0.1% crystal violet as described [32]. Based on the results shown in Fig. S1A, 10 µg/ml cyclic RDG (GD25β1 cells) or 25 µg/ml (BJ hTERT cells) was used for experiments on FN where indicated. Error bars represent standard deviation.

### Attachment signaling assay

The attachment assay was performed in serum-free DMEM on 6-well plates coated with FN, VN or PL with serum-starved cells as described [37] for 30 minutes unless otherwise indicated. Importantly, the cells were allowed to recover in suspension for one hour at 37°C before the cells were seeded, and any additives were given during this resting period 20 minutes prior to seeding. All cells in each well (adherent+non-adherent) were collected, solubilized with SDS sample buffer and analyzed by western blotting.

### Cell stretching assay

Stretching of cells in serum-free DMEM was performed in silicon chambers attached to a stretch apparatus exerting uniaxial cyclic stretch with adjustable displacement (built in-house). Five chambers can be stretched in parallel in the apparatus (Fig. S1B).

Silicone chambers with 2×2 cm^2^ inner dimensions were cast and new bottoms were attached for each experiment, since we were unable to obtain reproducible results when re-using the chambers in spite of extensive washing according to protocols available in publications or recommended by silicone chamber distributors. Chambers with newly attached bottoms were washed in 70% ethanol and at least 4 times with sterile water before use. Subsequently, the chambers were coated with 20 µg/ml FN or VN for three hours at room temperature (RT) under tilting. Any remaining non-coated surface was blocked using 1% heat-treated BSA over night at 4°C, and the chambers were then washed twice with PBS.

Before seeding, the cells were treated as described under “Attachment signaling assay”, except for the omission of the resting period in suspension. In each chamber, 60000 BJ hTERT cells or 225000 GD25β1 cells were seeded and allowed to spread for three hours. This period was chosen to minimize the deposition of cell-derived ECM proteins and still allow the initial peak in phosphorylation reactions during cell attachment to decline.

The cells were stretched for 10 minutes (uniaxial cyclic stretch 20%, 0.4 Hz) in the cell culture incubator. The stretching was stopped while the chambers were in the stretched position and the cells were harvested with SDS sample buffer. The samples were then analyzed by western blotting.

### SDS-PAGE and western blotting

Cell lysates were resolved by SDS-PAGE on BioRad pre-cast mini-gels. The separated proteins were transferred to Hybond-ECL nitrocellulose membrane (GE Healthcare, UK) and probed for different antigens as indicated. ECL signals were captured on x-ray films and scanned with a minimum resolution of 350 dpi or recorded with a BioRad ChemiDoc™ MP System.

Quantification of western blots was performed according to the “ImageJ method version 2” (http://www.lukemiller.org/journal/2007/08/quantifying-western-blots-without.html). Further calculations comprised the normalization of the actin signal by setting the actin signal of the stimulated sample (without inhibitors) to 1. These normalized actin values were then used to adjust the signal intensities derived from the respective antibody according to the loading. Error bars represent standard error of the mean (s.e.m.). The two-tailed Student's t-test was used for statistical comparisons and p<0.05 was considered as statistically significant.

### Total internal reflection fluorescence microscopy and image analysis

Cell spreading kinetics was monitored with total internal reflection fluorescence (TIRF) microscopy. The TIRF setup consisting of an E600FN upright microscope (Nikon, Kanagawa, Japan) and a climate box thermostated to 37°C was described earlier [37]. Invasin-coated cover slips were treated with BSA to block uncoated surface and were then used as bottoms of an open 200 µl chamber. Cells were labeled with the dye Vybrant DiO (Molecular Probes) and the fluorescence of adhering cells was monitored by excitation with an argon laser (ALC60X, Creative Laser Production, Munich, Germany) at 488 nm and detection at 525/25 nm. The membrane spreading process was imaged with an Orca-ER camera under control of the MetaFluor software (Molecular Devices Corp, Downington, PA). Images were acquired every 5 s using exposure times up to 200 ms and with the laser beam blocked between captures.

The cell contact area has previously been analyzed one cell at a time using the ImageJ software [37]. Here, we developed a new method based on image foresting transform (IFT, [38]). IFT is a graph- and seed-based method [39], where the image is interpreted as a graph and each image element corresponds to a node in a graph. A number of points known to belong to a certain class, so-called seeds, are initially marked manually by the user prior to the segmentation. The image is then segmented by assigning the label of the closest seed-point to each node, determined by the minimum cost path. The image segmentation, i.e. the classification of the image into cell and background, is crucial for the further analysis and not trivial because of the varying fluorescence intensities in our assay.

To facilitate the analysis, a stand-alone Matlab application with a graphical user interface has been developed. The user marks the seed-points by clicking on the cells with a unique label in one or several slices of the image volume and similarly marks the background. The IFT method classifies the cells and assigns a label to each one of the cells. Based on the segmentation results, the area of each cell is quantified for each slice and can be exported as a csv file. Graph preparations were made using the IgorPro software (WaveMetrics Inc, Lake Oswego, OR). Traces are presented as mean area ± standard error of mean.

### Measurements of intracellular ATP

a) Single cell analysis: GD25β1 cells were transfected to transiently express the fluorescent ATP/ADP reporter protein Perceval [40], [41] and cytosolic mCherry as an internal standard (pmCherry-C1, #632524, Clontech). For transfection, electroporation using the AMAXA system was used as described earlier [37]. 0.5 µg of each plasmid (encoding Perceval and mCherry) was used per transfection. The cells, cultured on glass coverslips in complete medium for 20 h after the transfection, were pre-incubated for at least 30 minutes at 37°C in experimental buffer (125 mM NaCl, 4.8 mM KCl, 1.2 mM MgCl_2_, 1.3 mM CaCl_2_, 25 mM HEPES, 5.56 mM glucose, pH set to 7.40 with NaOH) before starting the measurements. When indicated, the cells were exposed to rotenone (1 µM) for approx. 45 min (similar to the integrin stimulation assays), and for the final ATP depleting phase, rotenone, sodium azide (650 µg/ml) and 2-deoxy-D-glucose (10 mM) were added to experimental buffer without glucose. The coverslips with the attached cells were used as exchangeable bottoms of an open 50 µl superfusion chamber, which was mounted on the stage of a confocal microscope (see below) and maintained at 37°C.

The average cytoplasmic concentration of ATP was analyzed with a confocal system (Yokogawa CSU-10 spinning disk, Andor Technology, Belfast, Northern Ireland) attached to an Eclipse TE2000microscope (Nikon, Kanagawa, Japan) with a 60×, 1.40-NA objective. Perceval and mCherry were alternately excited at 488 nm by an argon ion laser (ALC60X, Creative Laser Production, Munich, Germany) and at 561 nm by a diode-pumped solid-state laser (Jive, Cobolt AB, Solna, Sweden), respectively, using an acousto-optic tunable filter (AA Optoelectronics, Orsay Cedex, France) for wavelength switching. Emission wavelengths were selected with the following filters (center wavelength/half-bandwidth): 527/27 nm for Perceval (Semrock, Rochester, NY, USA) and 645 nm longpass for mCherry (Melles Griot, Didam, The Netherlands). Fluorescence was detected with an EMCCD camera (DU-888E, Andor Technology, Belfast, Nothern Ireland) under MetaFluor software control (Molecular Devices Corp., Downingtown, PA, USA Perceval/mCherry image pairs were acquired every 5 s.

Image analysis was performed using the FIJI (http://fiji.sc/wiki/index.php/Fiji) software. The initial Perceval/mCherry fluorescence ratio was normalized to unity to compensate for variability in expression levels (R/R_0_). Igor Pro (Wavemetrics, Lake Oswego, OR) software was used for slope correction and illustrations. Further analysis was performed manually and in Excel.

b) Cell population analysis: ATP measurements in 96-well plate assay format of serum-starved control- or rotenone-treated cells were performed with the ATP assay kit from BioVision according to the manufacturer's manual. The results were expressed as percentage of the ATP levels in control cells. The error bars represent standard deviation.

### Total antioxidant capacity

The total antioxidant capacity (TAC) of BJ hTERT and GD25β1 cells (comprising both enzymes/proteins and small molecule antioxidants) was determined with the kit from BioVision using Trolox to standardize antioxidants according to the manufacturer's instructions. The error bars represent standard deviation and the two-tailed Student's t-test was used for statistical comparisons. p<0.05 was considered statistically significant.

### Superoxide measurement

Measurements investigating any possible superoxide scavenging effect of ascorbate and Asc-2P using the xanthine/xanthine oxidase system were performed as described by Benov et al. [42]. A Tecan infinite M200 plate reader was used to detect the fluorescence of ethidium (generated from hydroethidine by superoxide) in a white 96-well plate with a total reaction volume of 100 µl. Excitation wavelength was 495 nm and emission was detected at 580 nm. The measurements were carried out at 23°C.

### Pro-oxidant measurements using 2′,7′-dihydrodichlorofluorescein diacetate (H_2_DCFDA)/2′,7′-dichlorofluorescein (DCF)

Cells stimulated through attachment as described above were loaded with 5 µM H_2_DCFDA 3 minutes before the end of the experiment as described [23]. The cells were lysed in 50 mM Tris buffer pH 7.4 containing 0.5% SDS, and fluorescence of DCF was analysed in duplicates in a black half-area 96-well plate by the Tecan infinite M200 plate reader at 23°C (excitation wavelength 490 nm, emission 520 nm).

## Results

### Early cell spreading generates contraction-independent signals

Myosin II-dependent contraction force is required for the growth of newly formed adhesion complexes [43]–[45]. To investigate the possibility that tension from such contraction is involved in the generation of signals during the early cell attachment and spreading reactions, we used two inhibitors targeting non-muscle myosin II (Blebbistatin) and the upstream regulator RHO-associated, coiled-coil containing kinase (ROCK; inhibitor Y27632) in attachment assays. Cells were seeded into wells coated with FN or the non-adhesive polymer Pluronic (PL), and in order to compare signals generated by αvβ3 and α5β1 and integrins after binding to FN, we used the fibroblast-like cell lines GD25 (lacking the β1 subunit) and GD25β1 (in the presence of cyclic RGD peptide to block αvβ3). In addition to these transformed cells, the non-transformed BJ hTERT cells were studied.

**Figure 1:**
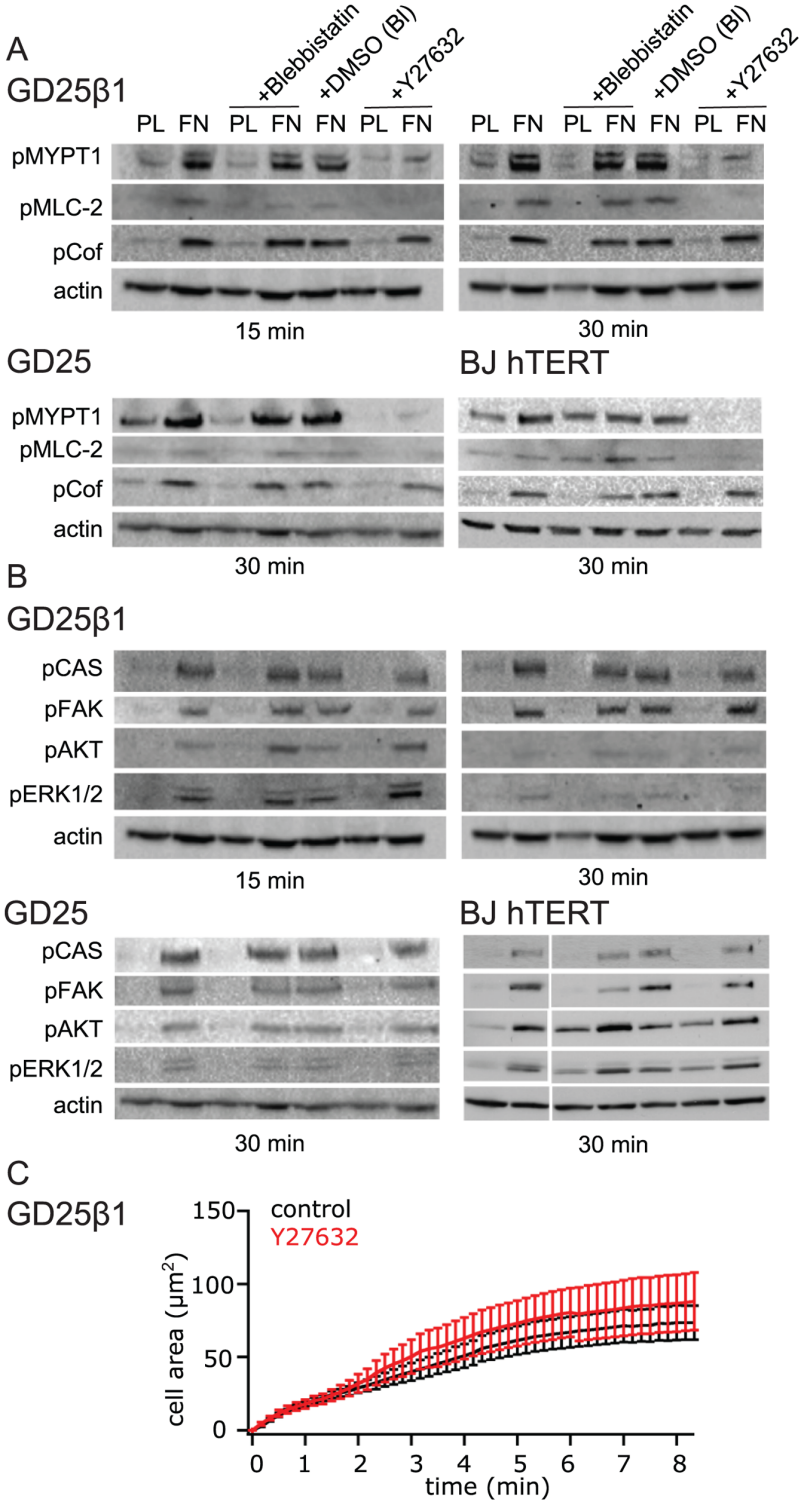
Effect of cell contraction inhibitors on selected known ROCK substrates (A) and “classical” integrin signals (B) after attachment of GD25β1 (n = 2), GD25 (n = 3) and BJ hTERT (n = 3) cells. The cells were treated with 100 µM of the non-muscle myosin inhibitor Blebbistatin, Blebbistatin vehicle control DMSO (DMSO (BL)), or 10 µM of the ROCK inhibitor Y27632 as indicated, and seeded on non-adhesive PL or FN (GD25) or FN in the presence of soluble cyclic RGD peptide (GD25β1 and BJ hTERT). After attachment for the indicated times, the cells were solubilized and subjected to western blot analysis. (**C**) TIRF recording of cell spreading kinetics on the β1 integrin-selective ligand invasin for GD25β1 control cells (black, n = 24) and GD25β1 cells treated with 10 µM ROCK inhibitor Y27632 (red, n = 17).

Three proteins involved in actomyosin regulation and known to be phosphorylated by ROCK were analyzed. Phosphorylation of myosin phosphatase targeting subunit 1 (MYPT1) at the inhibitory site T853 was prominently induced early during cell attachment, and as expected it was abolished almost completely by the ROCK inhibitor Y27632 (Fig. 1A). In addition, Y27632 markedly reduced the stimulatory phosphorylation at S19 of myosin light chain (MLC) in the investigated cell lines. Since MLC S19 can also be phosphorylated by other kinases than ROCK, primarily by MLC kinase, the effect by Y27632 on MLC pS19 levels may partly be ascribed to the increased MYPT1 activity. The attachment-induced phosphorylation of cofilin S3, i.e. the inhibition of its actin filament severing activity, was not markedly reduced by Y27632 (Fig. 1A). This result indicates that cofilin S3 was phosphorylated by a ROCK-independent pathway at this early stage of cell adhesion, possibly by PAK downstream of RAC [46].

While Y27631 had strong effects on known ROCK targets, it did not reduce attachment-induced phosphorylation of several other well-known integrin-regulated proteins such as ERK, AKT, and p130CAS (Figs. 1B). Neither did Y27632 inhibit the early membrane spreading reaction of GD25β1 (Fig. 1C) and BJ hTERT cells (not shown) on the β1 integrin-selective ligand invasin as recorded by TIRF microscopy. In this assay, the integrin signal is detected almost immediately after ligand binding. Direct inhibition of myosin II by Blebbistatin confirmed the Y27632 results, i.e. the phosphorylation levels of ERK, AKT, and p130CAS were not affected (Figs. 1B). The results demonstrate that these reactions occurred independently of myosin II-dependent force acting on the integrins or integrin-associated proteins at this stage of attachment. Blebbistatin reduced the phosphorylation level of focal adhesion kinase (FAK) Y397 in BJ hTERT cells but not in GD25β1 or GD25, indicating that intracellular contraction can influence the regulation of this site in a context-dependent manner (Fig. 1B). Results from cell attachment to FN via either integrin α5β1 or αvβ3 were highly similar (Fig. 1A, B).

### Signals induced by cell attachment and external force are different

**Figure 2:**
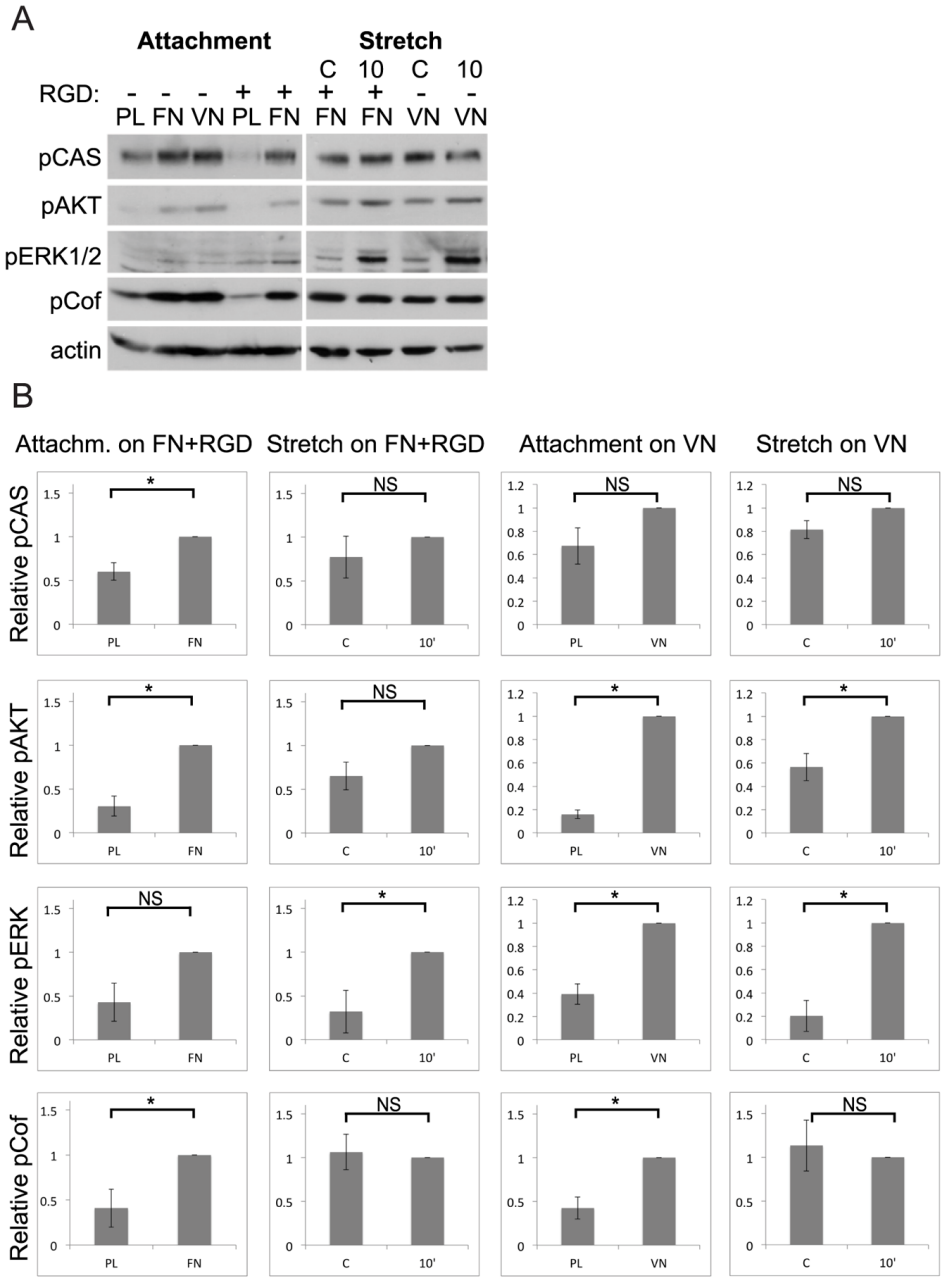
Comparison of GD25β1 signaling after attachment and cell stretching. Attachment and stretch assays were performed as described in Materials and Methods. C = non-stretched control; 10 = 10 minutes stretching. (**A**) Cell lysates were subjected to SDS-PAGE and analyzed by western blotting using antibodies against the indicated proteins. (**B**) Western blot signals of independent attachment and stretch experiments were quantified (attachment on FN+RGD: n = 3; stretch on FN+RGD: n = 4; attachment on VN: n = 4; stretch on VN: n = 4). Error bars represent s.e.m. * p<0.05; NS not significant.

In order to compare the integrin signals induced by cell attachment and external mechanical force, the cells were subjected to attachment and stretch assays in parallel on FN and VN. Consistent with the results in Fig. 1, the GD25β1 cells responded with robust elevated phosphorylation levels of the analyzed proteins to attachment and spreading on both substrates (via integrin α5β1 and αvβ3, respectively) for 30 minutes (Fig. 2).

Comparing cyclic stretching with non-stretched controls (C), the cells showed a robust phosphorylation of ERK1/2 with a peak after 10 minutes stretching, while no or smaller effects on the phosphorylation levels of AKT, p130CAS, and cofilin were seen (Fig. 2). FAK pY397 and MYPT1 pT853 levels were essentially unaffected after 10 min stretching (Fig. S2A,B). Cell stretching increased pERK also in BJ hTERT cells as exemplified in Fig. S2C, while the phosphorylation levels of the other proteins did not change (data not shown). The increase of pERK1/2 was not significantly affected by the presence of Blebbistatin or the ROCK inhibitor Y27632 in BJ hTERT cells (Fig. S2D) or GD25β1 (not shown). No major difference in stretch signals was observed when the two cell lines adhered via α5β1 or αvβ3 integrins, and this conclusion was supported by the assays performed with GD25 cells lacking β1 expression on FN and VN (Fig. S2E). Therefore, the subsequent experiments focused on β1 integrins.

These results show that cell stretching did not induce the same set of phosphorylation reactions as cell attachment. Although the basal phosphorylation levels were higher in the stretch experiments since the cells were adherent, they did not represent maximal levels (present approximately one hour after seeding, data not shown). The phosphorylation of ERK1/2 appears to be a major tension-responsive signal

### Mitochondrial ROS affects AKT and ERK1/2 during cell attachment

The possible effects of ROS on the signaling pathways induced by the two distinct types of integrin stimulation was investigated by several approaches. Using the commercial ROS sensor H_2_DCFDA we detected an increase in fluorescence in response to 30 minutes attachment in GD25β1 cells (data not shown) in agreement with other reports (refs). However, since H_2_DCFDA has been found to measure pro-oxidative processes in general rather than specifically H_2_O_2_ or other types of ROS [31], [47], [48], we chose to focus on other methods. First, we studied the contribution of mitochondrial ROS to the signaling responses using rotenone as a specific inhibitor of complex I in the respiratory chain. The compound was used at 1 µM, a commonly used concentration for rapid effects in short-term experiments [16].

**Figure 3:**
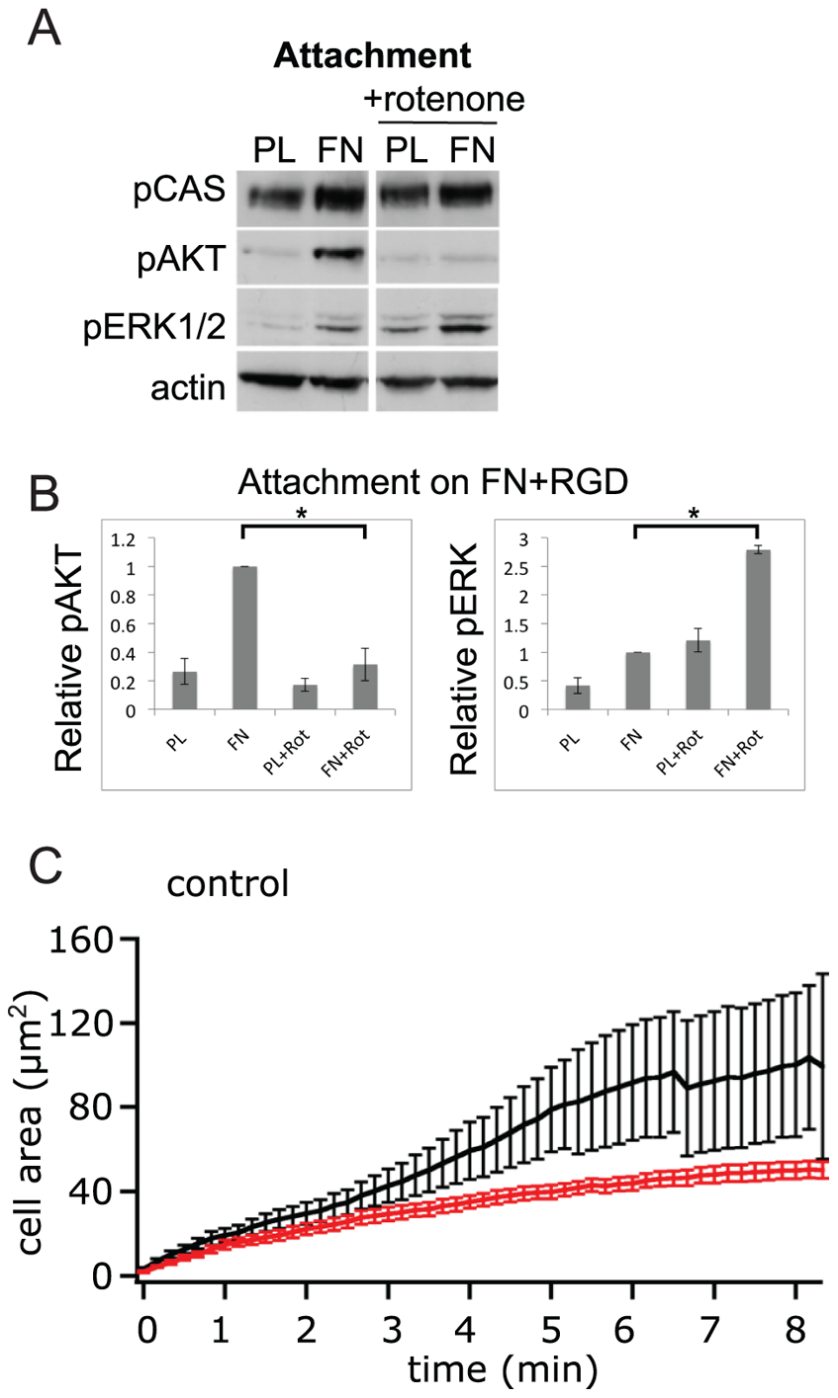
Effect of rotenone on GD25β1 signaling during attachment. The cells were treated with 1 µM rotenone where indicated and seeded on non-adhesive PL or FN in the presence of cyclic RGD peptide in the medium. (**A**) A representative western blot for attachment is shown. (**B**) Western blot signals of three independent experiments were quantified. (**C**) TIRF recording of cell spreading kinetics on the β1 integrin-selective ligand invasin for GD25β1 control cells (black, n = 9) and GD25β1 cells treated with 1 µM rotenone (red, n = 10). Error bars represent s.e.m. * p<0.05; NS not significant.

**Figure 4:**
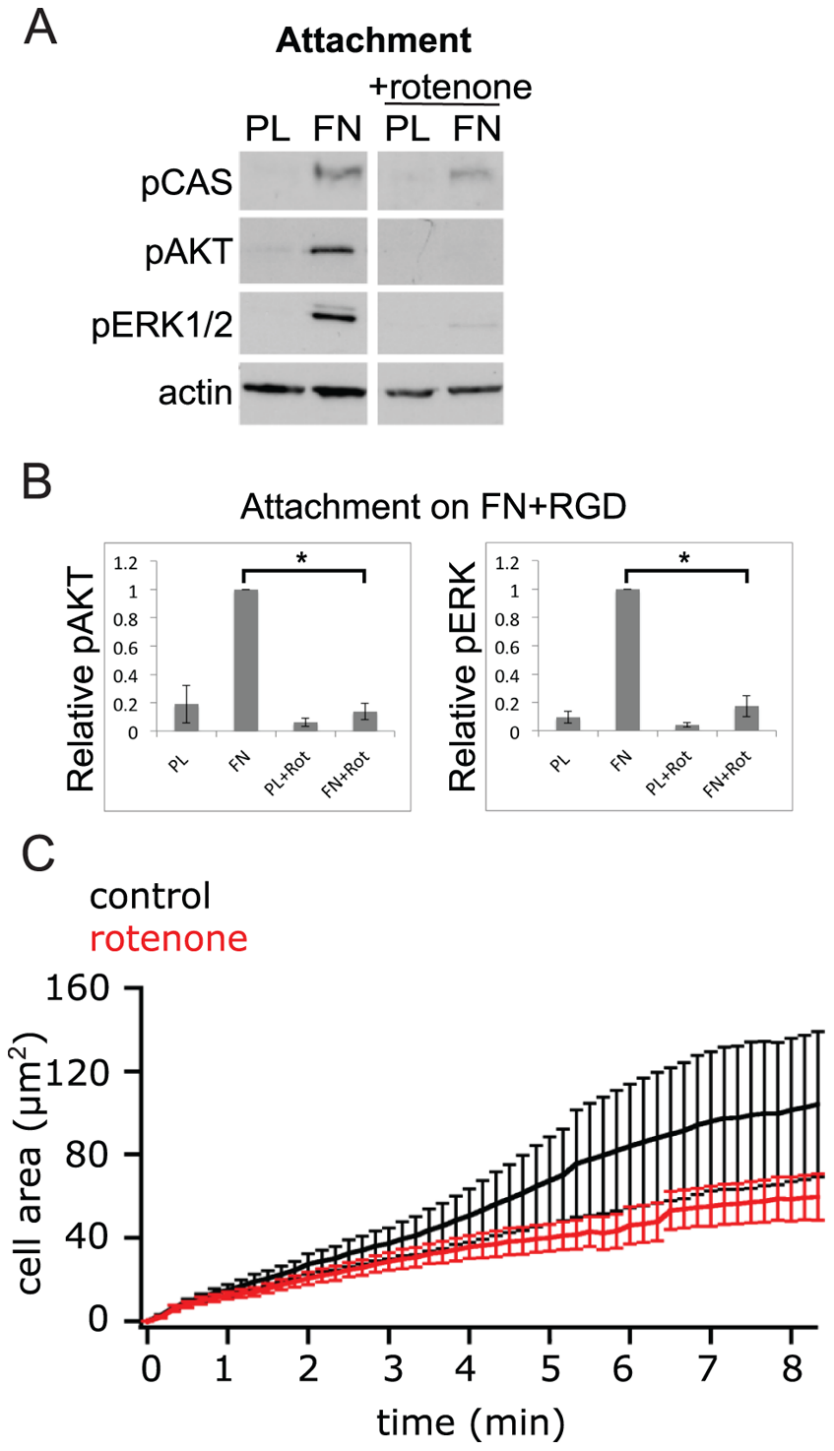
Effect of rotenone on BJ hTERT signaling during attachment. The cells were treated with 1 µM rotenone where indicated and seeded on non-adhesive PL or FN in the presence of cyclic RGD peptide in the medium. (**A**) A representative western blot for attachment is shown. (**B**) Western blot signals of three independent experiments were quantified. (**C**) TIRF recording of cell spreading kinetics on the β1 integrin-selective ligand invasin for BJ hTERT control cells (black, n = 10) and BJ hTERT cells treated with 1 µM rotenone (red, n = 11). Error bars represent s.e.m. * p<0.05; NS not significant.

Administration of rotenone efficiently inhibited the activation of AKT in response to attachment in both investigated cell lines (Figs. 3, 4). In BJ hTERT cells (Fig. 4), it also abolished the attachment-induced phosphorylation of ERK1/2 whereas it led to elevated pERK1/2 levels in GD25β1 cells (Fig. 3). Rotenone clearly reduced the rate of the initial membrane spreading reaction of GD25β1 on the β1 integrin-selective ligand invasin as recorded by TIRF microscopy (Fig. 3C) and the same trend was observed for BJ hTERT cells (Fig. 4C). The effect on membrane spreading, a process driven by actin polymerization [37] was detectable within 3 minutes after ligand binding. In a related fashion, rotenone treatment reduced the number of adhering cells in conventional adhesion assays (Fig. S3), apparently because rotenone-treated cells were spreading slower than control cells and were therefore more prone to be washed away. Importantly, those less spread cells were still able to generate certain signals of comparable or even higher intensity as non-treated cells (Fig. 3). Rotenone treatment of cells subjected to cyclic stretch did not significantly affect the analyzed signaling reactions (data not shown) and no cell detachment from the silicone substrate was observed.

**Figure 5:**
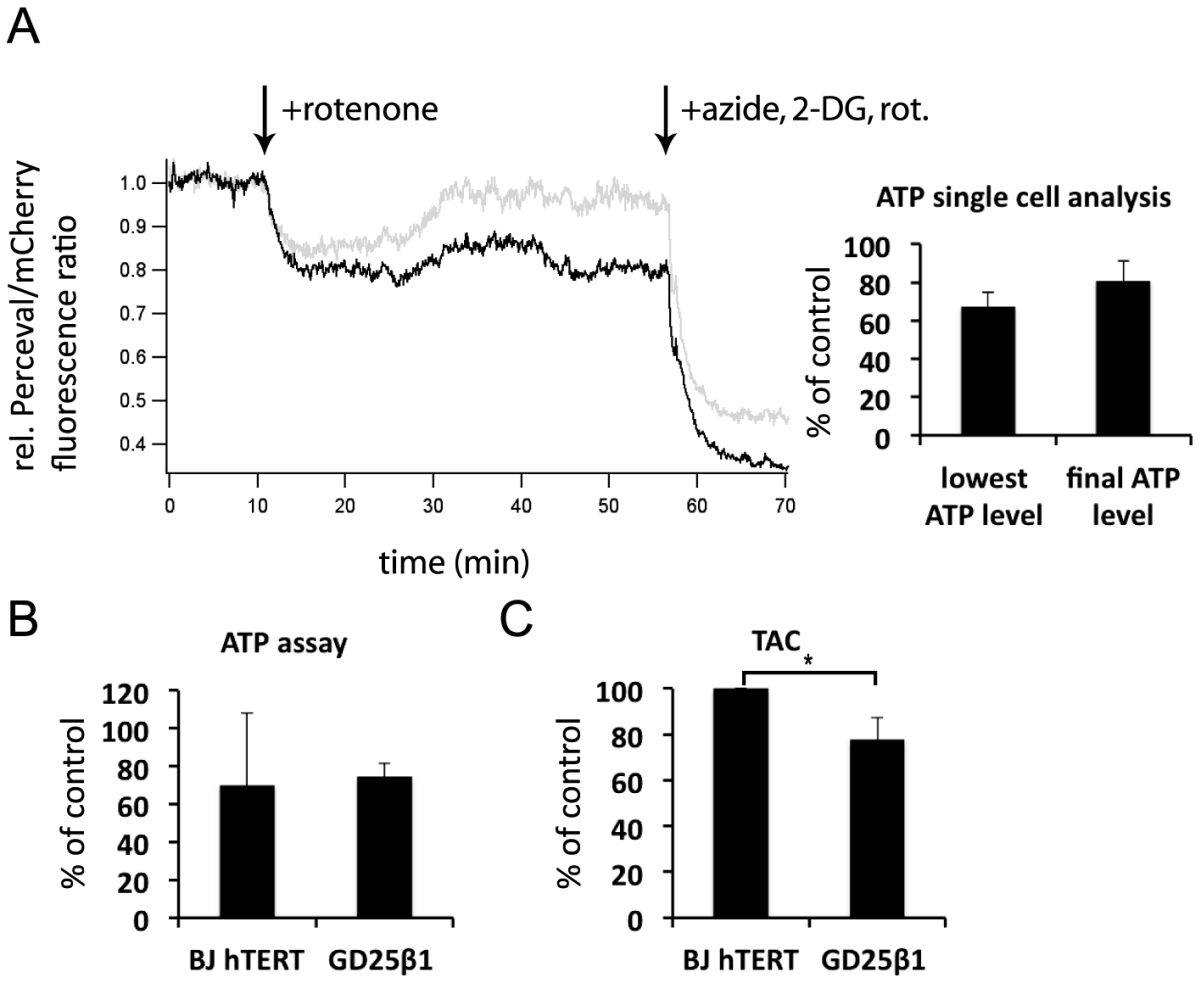
Measurements of intracellular ATP and antioxidant capacity. (**A**) Confocal microscopy recordings of ATP in single cells. GD25β1 cells were transfected with Perceval and prepared as described in Material and Methods. The traces are representative recordings of two cells. The addition of 1 µM rotenone and the subsequent treatment with a combination of rotenone, sodium azide and the glycolysis inhibitor 2-deoxy-D-glucose (2-DG) to maximally reduce ATP levels are indicated by the arrows. Quantifications from 8 cells are shown to the right. The bars show means of the lowest ATP level during treatment with rotenone alone and the ATP level just before addition of the inhibitor combination. (**B**) ATP levels of serum-starved BJ hTERT and GD25β1 cells after 45 min treatment with rotenone as determined by a colorimetric ATP assay performed described in Materials and Methods. n = 2. (**C**) Total antioxidant capacity (TAC) of serum-starved BJ hTERT and GD25β1 cells as determined by a colorimetric TAC assay described in Materials and Methods. The results are expressed as Trolox equivalents and were normalized to BJ hTERT cells in order to be able to compare the two cell lines with each other. n = 3. All error bars in Fig. 5 represent standard deviation, * p<0.05.

The possibility that the observed rotenone effects were caused by ATP depletion was investigated by two different methods. The kinetics of ATP level changes in single cells were measured using the fluorescent reporter protein Perceval [40]. The ATP level was rapidly reduced by rotenone to an average level of approximately 67% of the level at the beginning of the analysis (Fig. 5A). After 45 minutes, most cells however showed recovered ATP levels up to approximately 80% of the original level. According to a colorimetric ATP assay for entire cell populations, rotenone treatment reduced ATP levels to about 70% and 75% in BJ hTERT and in GD25β1 cells, respectively (Fig. 5B). Thus, the reduced phosphorylation of AKT and ERK in the presence of rotenone were most likely not due to ATP deficiency.

As cell lines have been reported to possess different antioxidant capacities, which can influence the outcome of experiments involving ROS, we determined the total antioxidant capacity (TAC) of the two cell lines used. A slightly, but significantly lower total antioxidant capacity of GD25β1 cells in comparison to BJ hTERT cells was measured with a colorimetric TAC kit (Fig. 5C).

### Asc-2P affects ERK1/2 during cell stretching

**Figure 6:**
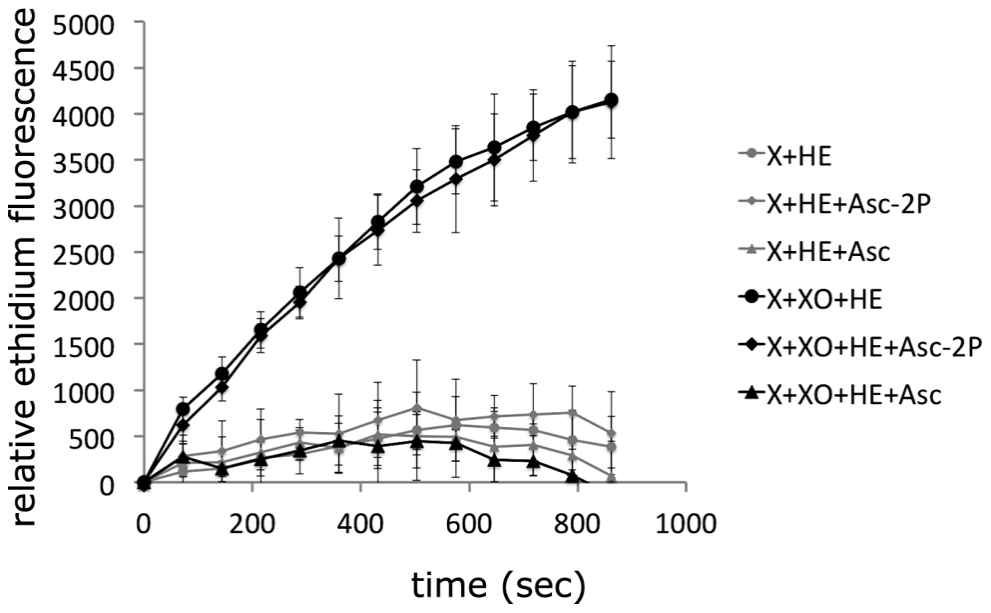
Analysis of superoxide-scavenging activity. The presence of superoxide generated by the xanthine (X)-xanthine oxidase (XO) system was measured by the fluorescence derived from superoxide-mediated conversion of non-fluorescent hydroethidine (HE) to fluorescent ethidium in the absence or presence of ascorbate (Asc) or its stable derivative L-ascorbic acid 2-phosphate sesquimagnesium salt hydrate (Asc-2P).

The effects of the natural antioxidant vitamin C (ascorbate) on integrin-stimulated responses were analyzed by use of a stable ascorbate derivative (L-ascorbic acid 2-phosphate sesquimagnesium salt hydrate, abbreviated Asc-2P) that is converted into ascorbate by cellular phosphatases. With the stable derivative, moderate levels of the labile ascorbate are continuously generated, which are considered to have antioxidant effects without the potential pro-oxidative effects described for high concentrations of ascorbate added to culture media [49]. In our serum-free conditions, Asc-2P is probably converted to ascorbate mostly intracellularly [50], [51], but also by extracellular phosphatases. Since Asc-2P has been shown to have direct scavenging activity by itself towards several organic radical compounds [52], we analyzed its reactivity with superoxide. Using the xanthine-xanthine oxidase system to generate superoxide under defined conditions, we found that the ascorbate derivative itself cannot act as superoxide scavenger (Fig. 6).

**Figure 7:**
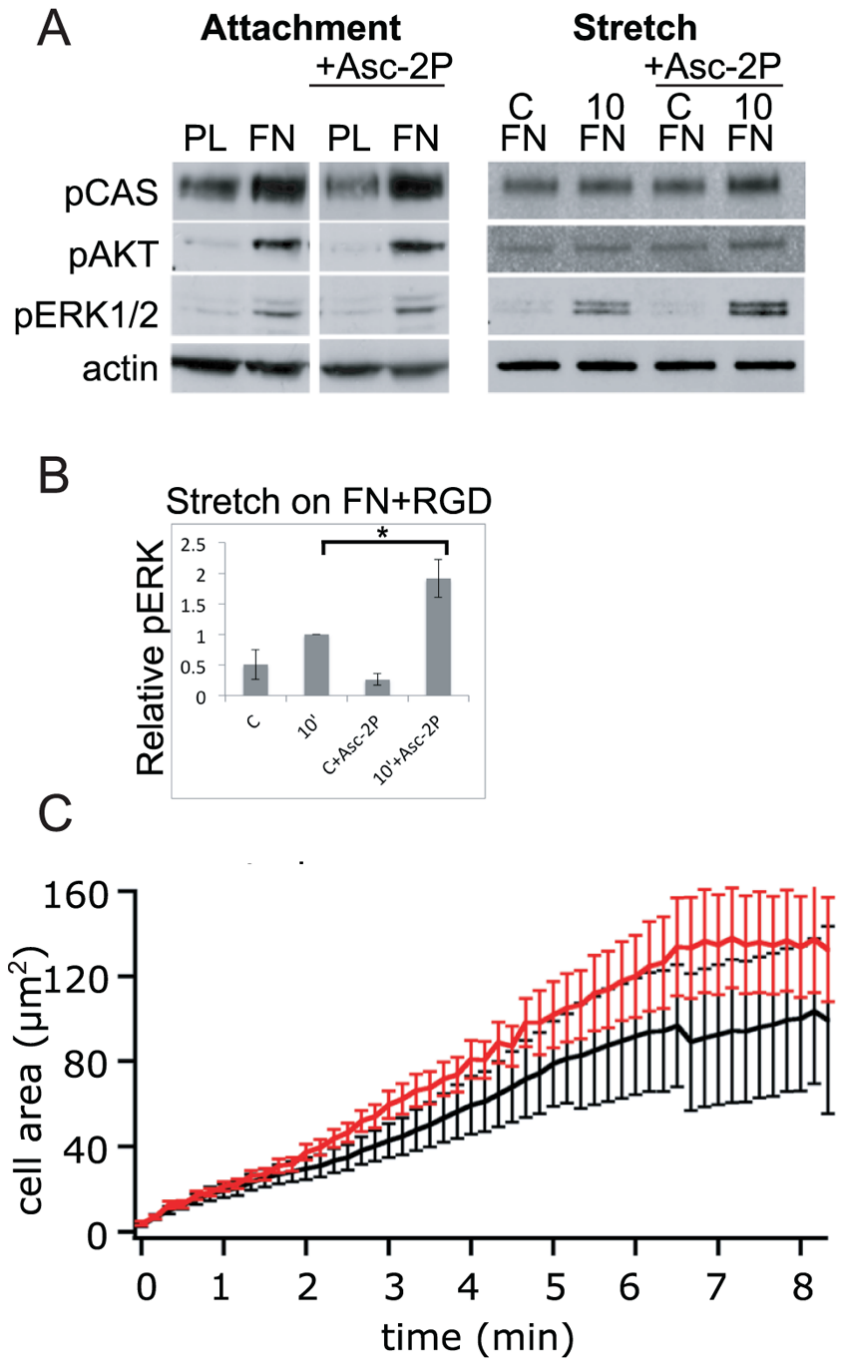
Effect of Asc-2P on GD25β1 signaling during attachment and cell stretching. C = non-stretched control; 10 = 10 minutes stretching. The cells were treated with Asc-2P where indicated and seeded on non-adhesive PL or FN in the presence of cyclic RGD peptide in the medium. (**A**) An example of a western blot. (**B**) Western blot signals of four independent experiments were quantified. Error bars represent s.e.m. * p<0.05; NS not significant. (**C**) TIRF recording of cell spreading kinetics on the β1 integrin-selective ligand invasin for GD25β1 control cells (black, n = 9) and GD25β1 cells treated with Asc-2P (red, n = 8). Another western blot analysis of Asc-2P-treated cells is shown in **Fig. S4A**.

Pre-incubation of cells with Asc-2P for 20 minutes did not significantly influence the analyzed phosphorylation reactions induced by attachment of GD25β1 (Fig. 7A) and BJ hTERT cells (data not shown). The rapid membrane spreading after the initial cell attachment was slightly enhanced by the Asc-2P treatment as analyzed by TIRF microscopy (Fig. 7C). However, Asc-2P led in GD25β1 cells to elevated ERK1/2 phosphorylation levels during cell stretching. Interestingly, this effect was primarily observed for ERK1/2 but somewhat variable (Fig. 7A, B, and Fig. S4A). The enhancement of pERK by Asc-2P was clear after stretching both via α5β1 and αvβ3 (Fig. S4A).

### Catalase affects ERK1/2 during cell stretching

Since ascorbate derived from Asc-2P may have exerted antioxidant effects both inside and outside of the plasma membrane, the source of the ERK1/2-regulating ROS during stretching was unclear.

**Figure 8:**
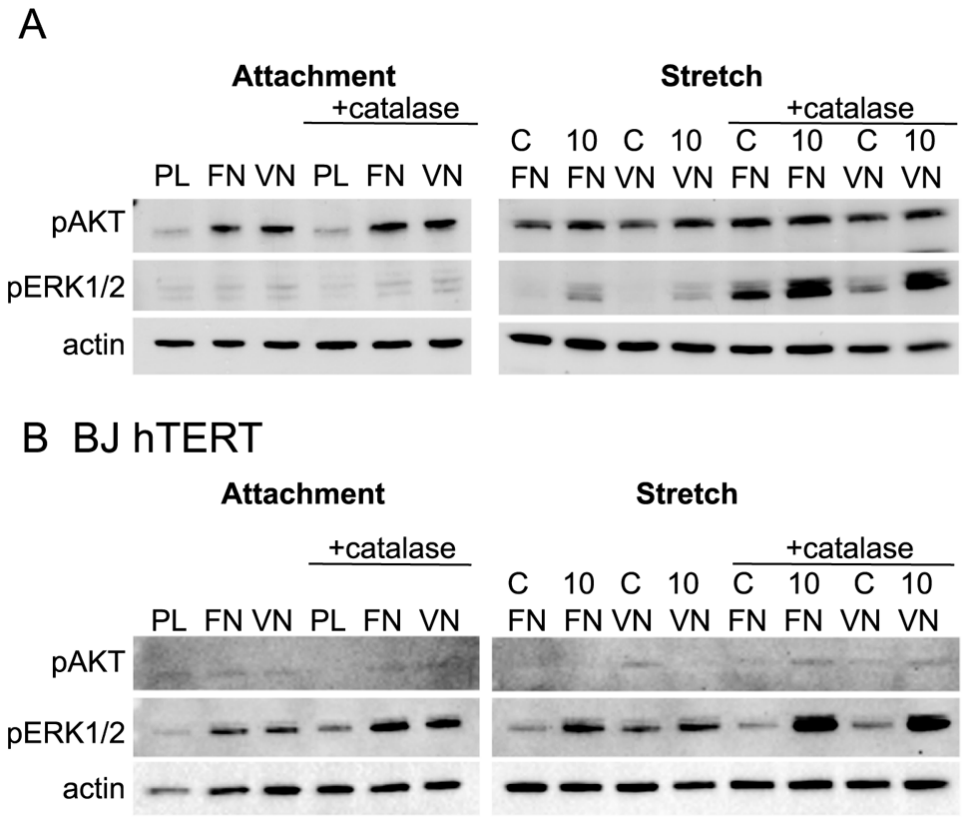
Effect of catalase on GD25β1 (n = 2) (A) and BJ hTERT (n = 2) (B) on signaling during attachment and cell stretching. C = non-stretched control; 10 = 10 minutes stretching. The cells were treated with 500 U/ml catalase where indicated and seeded on PL (non-adhesive) or FN in the presence of cyclic RGD peptide in the medium. More experiments were conducted in a similar fashion (e.g. Fig. S4B) with similar results.

To investigate the contribution of extracellular ROS, likely generated by NOXes located at the plasma membrane, catalase was added to the culture medium 20 minutes prior to integrin stimulation. The presence of catalase resulted in strikingly similar responses as Asc-2P: it selectively promoted stretch-induced accumulation of phosphorylated ERK1/2 in GD25β1 and BJ hTERT cells, but it did not markedly affect attachment-induced responses (Fig. 8A, B). Similar results were obtained when catalase was added together with superoxide dismutase (SOD) to the cells (Fig. S4B). We also tested a second batch of the enzyme and passed it through a protein desalting spin column in order to exclude that the stimulatory effects we observed were caused by any free transition metal ions (e.g. iron ions) in the catalase preparation, but obtained similar results (data not shown).

## Discussion

The mechanism(s) by which integrins generate intracellular signals is still unclear. Many downstream signaling pathways have been extensively characterized, but the initial molecular events linking integrins to these pathways are not known. However, based on previous studies two general principles for the generation of “integrin signals” have emerged: 1) Extracellular ligand-induced structural alterations in integrin cytoplasmic domains; this could potentially be achieved by receptor clustering and/or transmembrane propagation of conformational changes in integrins [8], [9]. 2) Mechanical force-induced structural alterations in integrin-associated proteins, or possibly in integrins themselves [2], [7], [53]. Our present study showed that integrin ligand-binding and externally applied cell stretching generated different signals and were affected by ROS from different sources.

### Signals induced by specific integrin stimuli

Phosphorylation levels of several proteins, including ERK1/2, p130CAS, FAK, AKT, MYPT1 and cofilin increased during the initial cell attachment and spreading phase, but of these proteins, only ERK1/2 was robustly phosphorylated after exposure of the cells to cyclic stretch. Notably, the ERK1/2 phosphorylation peaked after approximately 10 minutes of stretching, indicating that the cells adapted to the situation via feed-back reactions. Similarly, transient activation of ERK after stretch-stimulation of cells was also observed by other groups [54], [55]. Numerous studies have focused on integrin-mediated activation of ERK and several different pathways have been suggested [56]. Since this study showed that the responses triggered by integrin-ligand interaction and by mechanical force are different, it appears plausible that distinct mechanisms are utilized for ERK phosphorylation in these situations, and that this may be one reason for the previous varying results.

While activation of ERK by mechanical force seems to be a consistent finding in several biological studies, diverging results have been reported concerning activation of AKT and FAK [57]–[60]. One cause for the variability is most likely the use of different cell systems. Here, we analyzed fibroblast-like cells, which are programmed to respond to different types of mechanical force *in vivo* than e.g. cardiac myocytes, endothelial cells and epithelial cells. Different stretch parameters (static, cyclic, uni-/biaxial, duration, amplitude) and other conditions, such as cell density might also be important factors. In the present study the cells were kept at low density during stretching in order to minimize signaling from stretch-sensitive cell-cell contacts [30], [61].

Several of the attachment-induced signals could occur independently of cellular contractility according to our data with the myosin II and ROCK inhibitors (Fig. 1). These results are consistent with reports showing that RHOA-dependent pathways are transiently suppressed via SRC-mediated phosphorylation of p190RHO-GAP initially after integrin-mediated attachment, and that the suppression of the RHOA-ROCK pathway is required in order for cell spreading to occur [62], [63]. Notably, FAK pY397 levels were differently affected by Blebbistatin in GD25β1 and BJ hTERT cells. The results with GD25β1 cells showed that contraction is not required for the unfolding whereby Y397 is exposed for auto-phosphorylation [64]. The reduced level of FAK pY397 in Blebbistatin-treated BJ hTERT cells may therefore be due to altered phosphatase activity rather than decreased auto-phosphorylation.

Cofilin as an actin-severing protein promotes actin reorganization and polymerization. The binding of cofilin to actin filaments required for this activity is inhibited by phosphorylation of cofilin S3 or binding of cofilin to phosphoinositol-4,5-bisphosphate (PIP2) at the membrane [46]. Therefore, the observed increase in phosphorylation in response to cell attachment and early spreading (Fig. 2) will promote the stabilization of actin filaments. Stretching for 10 minutes did not lead to consistent major changes of the cofilin pS3 pool, indicating that the cytoskeletal rearrangements reported to follow upon stretching of cells [59] did not require increased cofilin activity in our cell lines.

p130CAS was recently shown to become tyrosine phosphorylated in a force-sensitive manner [5], [65]. Since phosphorylation of p130CAS is generally considered to be SRC family kinase-dependent [66], [67], and SRC activity is not increased in response to force [5] (consistent with our analysis, data not shown), force was concluded to promote p130CAS phosphorylation by exposure of potential acceptor sites in the protein. However, in our study the activation of p130CAS was only moderately elevated after cyclic stretch, as measured by phosphorylation at Y410 in the substrate domain. A possible explanation for the weak response could be that p130CAS exists in a “pre-extended” state in spread cells, which already allows a certain amount of phosphorylation in its substrate domain, as suggested by Sawada et al. [5].

### Role of mitochondrial ROS

In order to compare the effect of mitochondrial ROS in attachment- and stretch-induced signaling, we used the well-characterized natural compound rotenone. Rotenone did not influence stretch-induced signaling significantly in any of the cell lines. During attachment of BJ hTERT cells, rotenone dramatically reduced AKT and ERK phosphorylation levels, and pAKT was abolished also in GD25β1 cells. This would be consistent with higher activity of PTEN and other phosphatases when ROS release from mitochondria is inhibited. We have recently shown that β1 integrins in MCF-7 cells preferentially activate the AKT2 isoform during attachment and spreading [68], and in this context it is interesting to note that AKT2 has been reported to colocalize with mitochondria [69]. It remains to clarify how integrins transfer signals to mitochondria, although both diffusible factors and physical coupling via actin filaments have been suggested [10], [16], [70].

Surprisingly, rotenone led to an elevated ERK phosphorylation level during attachment of GD25β1 cells. Possibly the opposed ERK response in the two cell lines is related to a higher antioxidant activity in BJ hTERT cells (Fig. 5C and [71]) or to differences in the effect of ROS on transformed and non-transformed cells [72], [73], in our case GD25β1 and BJ hTERT cells, respectively [33], [74]. Blockage of complex I reduces the leakage of electrons from complex III into the intermembrane space and thereby decreases the amount of hydrogen peroxide that can diffuse into the cytoplasm [75], [76]. At the same time, rotenone increases the electron leakage from complex I into the mitochondrial matrix [75]. Though local antioxidant systems capture ROS arising there, cells likely differ in this ability, which may explain the reported contradictory effects of rotenone in different cell lines [17], [77]–[79]. While a reduced overall release of hydrogen peroxide into the cytoplasma from mitochondria by rotenone probably had direct effects on the AKT phosphorylation levels in both our cell lines as discussed above, it is conceivable that reduced hydrogen peroxide concentrations in the cells may cause diverging effects on ERK phosphorylation considering the complex regulation of this pathway. Since the pERK response to rotenone in GD25β1 may reflect a property of transformed cells [72], [73] it would be of great interest to identify the responsible ROS-regulated component. Importantly, the effects of rotenone on AKT and ERK were most likely not caused by a lack of ATP, since we (Fig. 5A, B) and others have shown that glycolysis maintains a sufficient level of ATP (around 70% of normal) in the presence of rotenone [77], [80].

### Effects of Asc-2P and catalase

In order to look at general ROS-mediated effects on signaling downstream of the two different integrin stimuli, we used a stable vitamin C derivative (Asc-2P). Vitamin C itself has a short half-life due to auto-oxidation and at high concentrations it can act as a pro-oxidant, a reaction that is further enhanced by transition metal ions [49], [50]. Other “antioxidants”, such as N-acetyl cysteine, are general “redox”-modulators rather than ROS scavengers [31] and therefore not suitable for our purpose. The use of Asc-2P that is converted to ascorbate by cellular phosphatases has been shown to provide long-term ROS scavenging activity [50], [51].

Administration of Asc-2P did not have any major effects on attachment-induced signaling, but a pronounced increase in ERK phosphorylation levels was seen in response to cell stretching. Strikingly, we saw a similar increase in pERK when scavenging hydrogen peroxide extracellularly with catalase. Vitamin C and catalase were previously reported to moderately trigger ERK phosphorylation in quiescent endothelial cells, and the authors suggested mitogen-like activities of vitamin C [81]. In that study no external force was applied, but actomyosin-dependent tension in the cell culture may have had a similar effect. While vitamin C could exert effects both in intra- and extracellular compartments, catalase added to the culture medium will presumably only act outside of the cells. Our observations suggest a negative role for extracellular hydrogen peroxide in stretch-induced ERK activation. Hydrogen peroxide, in contrast to other charged ROS molecules, can efficiently pass cell membranes through aquaporins and affect intracellular signaling reactions [31], [82]. One described source for extracellular hydrogen peroxide is superoxide produced by cell surface-located NOX.

Exogenous administration of hydrogen peroxide (0.5 mM) was recently shown to promote actin polymerization in the lamellipodia of PtK1 epithelial cells by a mechanism involving elevated ERK activity [83]. In our study we found that Asc-2P administration had rather the opposite effect on GD25β1 cells, namely a trend to an enhanced membrane spreading rate when ROS levels were reduced (Fig. 7C). On the other hand, reducing mitochondria-derived hydrogen peroxide by rotenone treatment decreased the initial membrane protrusion in both GD25β1 and BJ hTERT cells (Figs. 3C and 4C). Thus, ROS produced at different sites (i.e. mitochondria or NOXes) affected the membrane spreading reaction differently in our fibroblasts. In contrast to the PtK1 epithelial cells, the effect of mitochondrial ROS on actin polymerization in GD25β1 cannot be linked to ERK activity since pERK levels were elevated while the membrane spreading rate was reduced by rotenone. One possible cause for this disparity could be the usage of different factors driving actin nucleation and polymerization in these cells.

### Conclusion

In summary, we show that integrin signals derived from cell attachment and mechanical stretch are distinct. In response to both stimuli, α5β1 and αvβ3 induced very similar phosphorylation reactions. Most of the investigated attachment-induced reactions, but not all, occurred independently of myosin II-mediated contraction. The results extend the classical model of hierarchical integrin signals induced by ligand binding and integrin clustering [8] to encompass responses from mechano-stimuli. Our studies additionally show that mitochondrial ROS and extracellular ROS affected signals triggered by these two integrin stimuli differently. Future detailed characterization of the specific signaling responses will be useful for understanding the various types of integrin stimuli a cell encounters *in vivo*.

## Supporting Information

Figure S1 **(A) Adhesion assays.** BJ hTERT cells or GD25 and GD25β1 cells were allowed to attach and spread on the indicated substrates for 60 min in the presence of the indicated concentrations of cyclic RDG peptide. After washing, remaining cells were stained with crystal violet and the absorbance was measured at 600 nm. RGD concentration was plotted against the average absorbance values of triplicates (BJ hTERT) or duplicates (GD25/GD25β1) calculated after subtraction of background absorbance. Error bars represent standard deviation. **(B) Photograph of the stretch apparatus equipped with three chambers**.
(TIF)

Figure S2 **(A) Stretch assays with GD25β1 cells: effect on pY397 FAK phosphorylation levels.** The assays were performed as described in Materials and Methods. Cell lysates were subjected to SDS-PAGE and analyzed by western blotting using antibodies against the indicated proteins. **(B) Stretch assays with GD25β1 cells: effect on pT853 MYPT1 phosphorylation levels.** The assays were performed as described in Materials and Methods. Cell lysates were subjected to SDS-PAGE and analyzed by western blotting using antibodies against the indicated proteins. **(C) Comparison of BJ hTERT pERK signaling after attachment and cell stretching.** C = non-stretched control; 10 = 10 minutes stretching. Cell lysates were subjected to SDS-PAGE and analyzed by western blotting using antibodies against the indicated proteins. Western blot signals of three independent experiments were quantified. Error bars represent s.e.m. * p<0.05; NS non significant. **(D) Stretch assays with BJ hTERT cells: effect of ROCK and myosin inhibitor treatment.** The assays were performed as described in Materials and Methods. Blebbistatin (100 µM) and the ROCK inhibitor Y27632 (10 µM) were added 15 min before starting the stretching. DMSO was used as a vehicle control for Blebbistatin. Cell lysates were subjected to SDS-PAGE and analyzed by western blotting using antibodies against the indicated proteins. n = 2. Similar results were obtained with GD25β1 cells (n = 3). **(E) Attachment and stretch assays with GD25 cells.** The assays were performed as described in Materials and Methods. Cell lysates were subjected to SDS-PAGE and analyzed by western blotting using antibodies against the indicated proteins.
(TIF)

Figure S3 **Adhesion assays.** BJ hTERT (**A**) or GD25β1 (**B**) cells were allowed to attach and spread on FN for 15, 30 or 60 min. After washing, remaining cells were stained with crystal violet and the absorbance was measured at 600 nm. Time was plotted against the average absorbance values of triplicates calculated after subtraction of background absorbance. Error bars represent standard deviation.
(TIF)

Figure S4 **(A) Stretch assays with GD25β1 cells: effect of Asc-2P.** Stretch assays were performed with GD25β1 cells as described in Materials and Methods. The cells were stretched on VN- and FN-coated silicon in the presence of Asc-2P as indicated. **(B) Stretch assay with GD25β1 cells: effect of superoxide dismutase (SOD) and catalase.** Stretch assays were performed with GD25β1 cells as described in Materials and Methods. SOD (100 U/ml) and catalase (500 U/ml) were added 20 min before stretching as indicated. The cells were seeded on FN in the presence of cyclic RGD peptide in the medium C = control; 10 = 10 minutes stretch.
(TIF)
