# Supplementary figures and images for: The Role of Mechanical Force and ROS in Integrin-Dependent Signals
Source: PLoS One. 2013 May 30;8(5):e64897. doi: 10.1371/journal.pone.0064897 (PMC3667809; doi:10.1371/journal.pone.0064897)

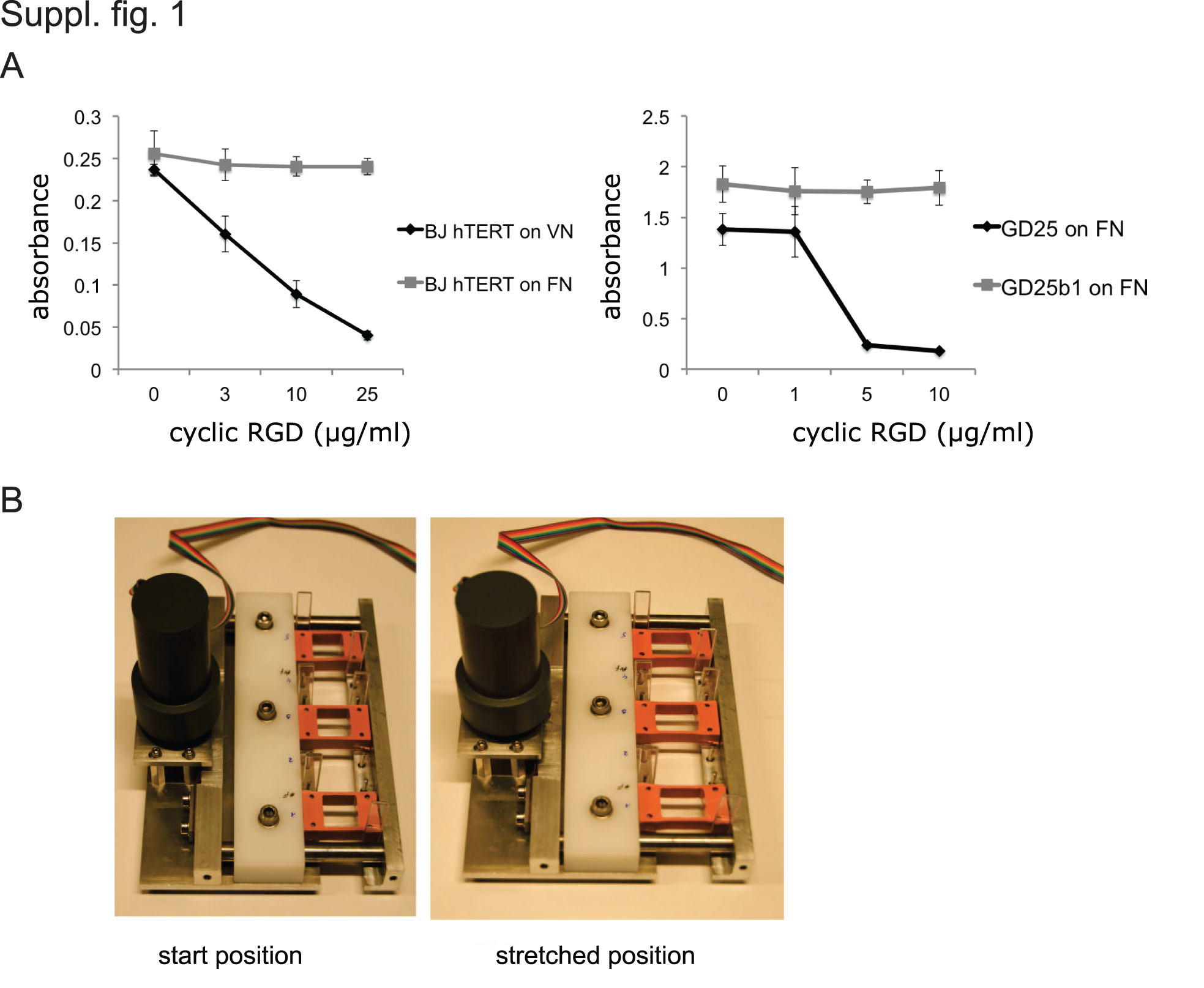

Supplement: Figure S1 — (A) Adhesion assays. BJ hTERT cells or GD25 and GD25β1 cells were allowed to attach and spread on the indicated substrates for 60 min in the presence of the indicated concentrations of cyclic RDG peptide. After washing, remaining cells were stained with crystal violet and the absorbance was measured at 600 nm. RGD concentration was plotted against the average absorbance values of triplicates (BJ hTERT) or duplicates (GD25/GD25β1) calculated after subtraction of background absorbance. Error bars represent standard deviation. (B) Photograph of the stretch apparatus equipped with three chambers. (TIF) [file pone.0064897.s001.tif]

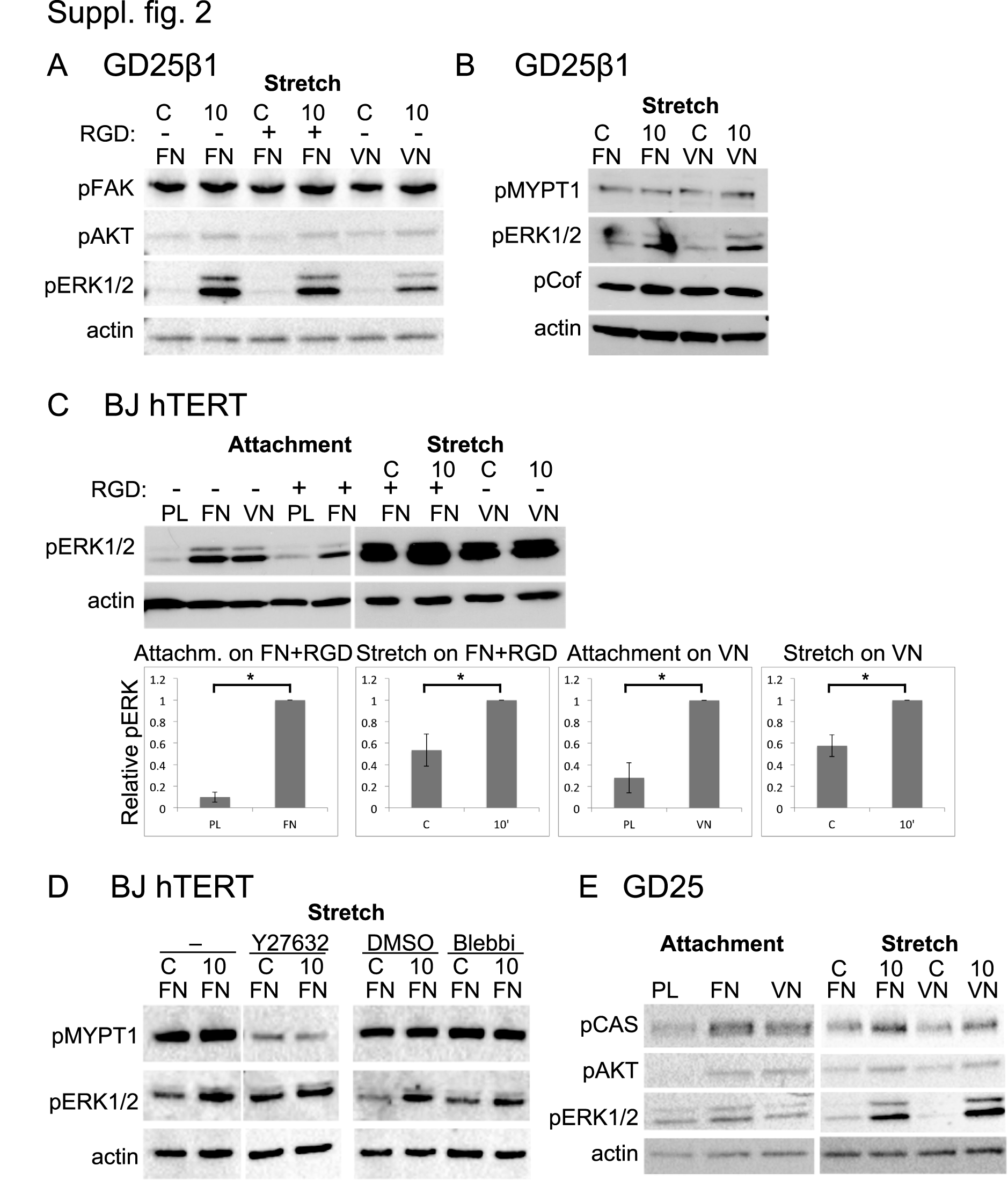

Supplement: Figure S2 — (A) Stretch assays with GD25β1 cells: effect on pY397 FAK phosphorylation levels. The assays were performed as described in Materials and Methods. Cell lysates were subjected to SDS-PAGE and analyzed by western blotting using antibodies against the indicated proteins. (B) Stretch assays with GD25β1 cells: effect on pT853 MYPT1 phosphorylation levels. The assays were performed as described in Materials and Methods. Cell lysates were subjected to SDS-PAGE and analyzed by western blotting using antibodies against the indicated proteins. (C) Comparison of BJ hTERT pERK signaling after attachment and cell stretching. C = non-stretched control; 10 = 10 minutes stretching. Cell lysates were subjected to SDS-PAGE and analyzed by western blotting using antibodies against the indicated proteins. Western blot signals of three independent experiments were quantified. Error bars represent s.e.m. * p<0.05; NS non significant. (D) Stretch assays with BJ hTERT cells: effect of ROCK and myosin inhibitor treatment. The assays were performed as described in Materials and Methods. Blebbistatin (100 µM) and the ROCK inhibitor Y27632 (10 µM) were added 15 min before starting the stretching. DMSO was used as a vehicle control for Blebbistatin. Cell lysates were subjected to SDS-PAGE and analyzed by western blotting using antibodies against the indicated proteins. n = 2. Similar results were obtained with GD25β1 cells (n = 3). (E) Attachment and stretch assays with GD25 cells. The assays were performed as described in Materials and Methods. Cell lysates were subjected to SDS-PAGE and analyzed by western blotting using antibodies against the indicated proteins. (TIF) [file pone.0064897.s002.tif]

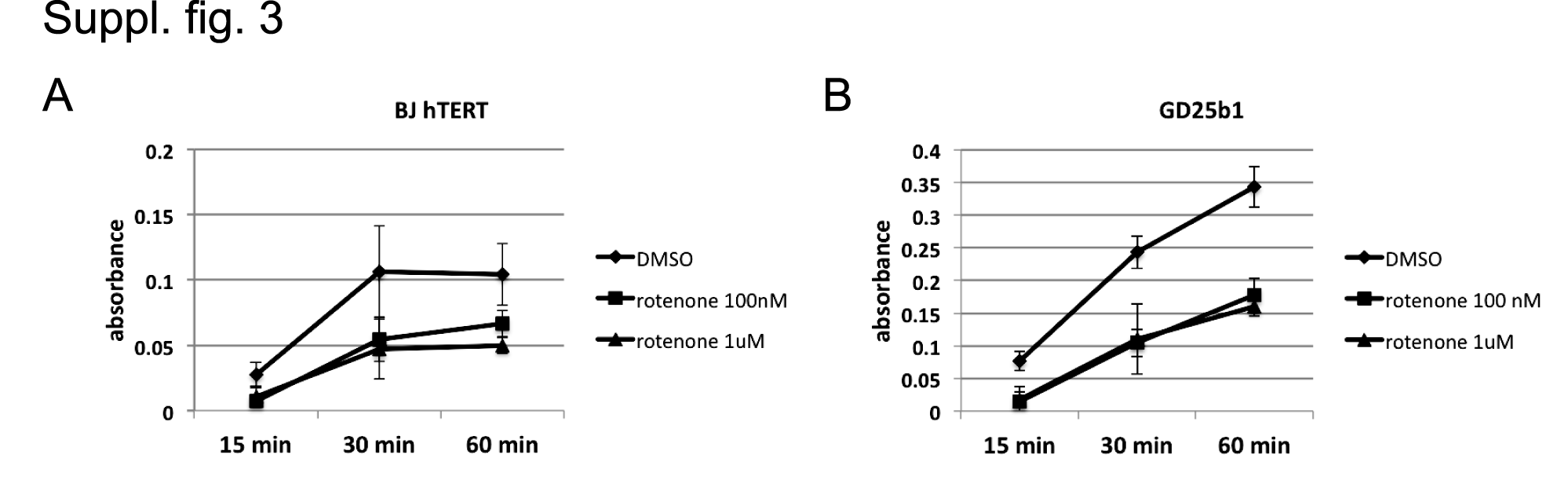

Supplement: Figure S3 — Adhesion assays. BJ hTERT (A) or GD25β1 (B) cells were allowed to attach and spread on FN for 15, 30 or 60 min. After washing, remaining cells were stained with crystal violet and the absorbance was measured at 600 nm. Time was plotted against the average absorbance values of triplicates calculated after subtraction of background absorbance. Error bars represent standard deviation. (TIF) [file pone.0064897.s003.tif]

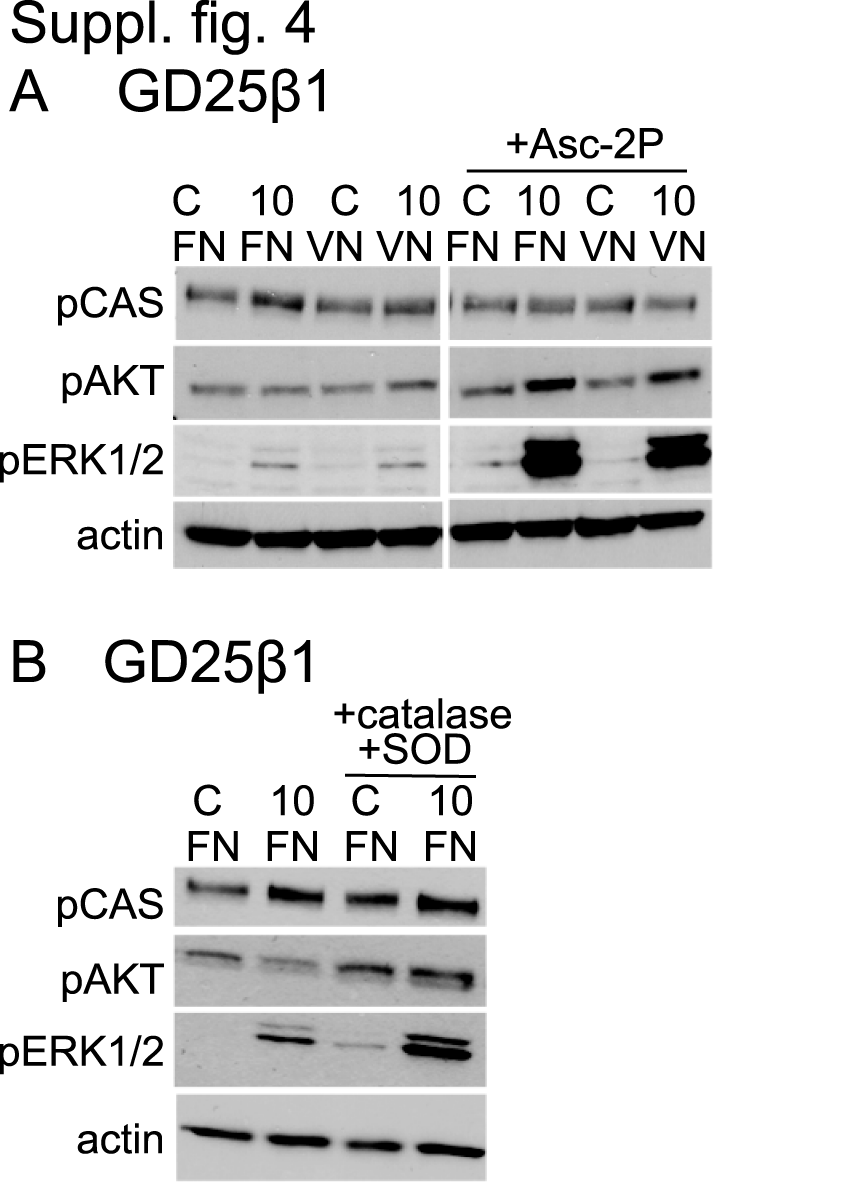

Supplement: Figure S4 — (A) Stretch assays with GD25β1 cells: effect of Asc-2P. Stretch assays were performed with GD25β1 cells as described in Materials and Methods. The cells were stretched on VN- and FN-coated silicon in the presence of Asc-2P as indicated. (B) Stretch assay with GD25β1 cells: effect of superoxide dismutase (SOD) and catalase. Stretch assays were performed with GD25β1 cells as described in Materials and Methods. SOD (100 U/ml) and catalase (500 U/ml) were added 20 min before stretching as indicated. The cells were seeded on FN in the presence of cyclic RGD peptide in the medium C = control; 10 = 10 minutes stretch. (TIF) [file pone.0064897.s004.tif]
